# Human uterine lymphocytes acquire a more experienced and tolerogenic phenotype during pregnancy

**DOI:** 10.1038/s41598-017-03191-0

**Published:** 2017-06-06

**Authors:** Dorien Feyaerts, Marilen Benner, Bram van Cranenbroek, Olivier W. H. van der Heijden, Irma Joosten, Renate G. van der Molen

**Affiliations:** 1 Department of Laboratory Medicine, Laboratory of Medical Immunology, Radboud University Medical Center, PO box 9101, 6500 HB Nijmegen, The Netherlands; 2 Department of Obstetrics and Gynaecology, Radboud University Medical Center, PO box 9101, 6500 HB Nijmegen, The Netherlands

## Abstract

Pregnancy requires a delicate immune balance that nurtures the allogeneic fetus, while maintaining reactivity against pathogens. Despite increasing knowledge, data is lacking on the transition of pre-pregnancy endometrial lymphocytes to a pregnancy state. Here, we immunophenotyped lymphocytes from endometrium (MMC), term decidua parietalis (DPMC), and PBMC for direct comparison. We found that the immune cell composition of MMC and DPMC clearly differ from each other, with less NK-cells, and more NKT-cells and T-cells in DPMC. An increased percentage of central memory and effector memory T-cells, and less naive T-cells in DPMC indicates that decidual T-cells are more experienced than endometrial T-cells. The increased percentage of CD4^+^CD25^high^CD127^−^ Treg in DPMC, including differentiated Treg, is indicative of a more experienced and tolerogenic environment during pregnancy. The Th cell composition of both MMC and DPMC was different from PBMC, with a preference for Th1 over Th2 in the uterine environment. Between MMC and DPMC, percentages of Th cell subsets did not differ significantly. Our results suggest that already before pregnancy a tightly controlled Th1/Th2/Th17 balance is present. These findings create opportunities to further investigate the underlying immune mechanism of pregnancy complications using menstrual blood as a source for endometrial lymphocytes.

## Introduction

Pregnancy requires a complex interplay of immune cells. Maternal lymphocytes need to accommodate the semi-allogeneic fetus and still maintain robust immune reactivity against pathogens. The barrier between the semi-allogeneic fetus and the maternal immune system is the placenta. At this fetal-maternal interface, maternal lymphocytes of the decidua come into close contact with cells of fetal origin, i.e. trophoblast cells. This contact occurs at two different sites, between invading trophoblast cells and the decidua basalis, which is the site of implantation, and chorionic trophoblast cells and the decidua parietalis, which are part of the membranes surrounding the fetus^1^. These trophoblast cells have restricted HLA expression (HLA-C, HLA-E, and HLA-G). Direct response to fetal allogeneic HLA is primarily via HLA-C, but also indirect presentation of fetal antigens by maternal APCs can elicit an anti-fetal maternal leukocyte response^2–6^. This restricted immune recognition makes that the uterine immune cell composition and phenotype is different from other mucosal sites^1^.

Each month, during the menstrual cycle, the uterus prepares itself for pregnancy by a large influx of leukocytes in the endometrium. When implantation takes place, the number of leukocytes increases even further. Without implantation, the endometrial lining and its leukocytes are shed during menstruation^7^. Natural killer (NK) cells are abundantly present in the human endometrium^8, 9^. Endometrial NK cells increase in number during the menstrual cycle, reaching a peak in the late secretory phase. If implantation occurs, endometrium will transform into decidua and the number of endometrial NK cells will increase even further and will make up 70% of the decidual leukocytes during the first trimester. These uterine NK cells are different from NK cells found in peripheral blood. They are characterized as being CD56^bright^CD16^−^, while NK cells found in peripheral blood are mainly CD56^dim^CD16^+^^8, 10^. Decidual NK cells produce specific cytokines and angiogenic factors to regulate invasion of fetal trophoblast cells and spiral artery remodeling^7, 10^.

Besides NK cells, also T cells are a major cell population in the endometrium and decidua^8, 11^. Decidual T cells differ from peripheral T cells by expression of activation markers such as CD45RO, CD69, HLA-DR, and CD25^12^, but their function and mechanism of fetus-specific immune recognition remains poorly defined^13^. It has long been thought that maternal tolerance towards fetal alloantigens was established by a predominance of T helper type 2 (Th2) immunity over Th1 immunity during pregnancy. However, this Th1/Th2 paradigm was found insufficient, since both Th1 and Th2 dominant immunity was observed in pregnancy complications^14^. Th17 cells produce IL-17 and mediate the induction of inflammation^15^. Higher levels of Th17 cells were found in women suffering from recurrent pregnancy loss and preterm delivery^16–18^. In contrast, mouse studies revealed that regulatory T cells (Treg) are essential for promoting immune tolerance towards the fetus, and activation of Treg is needed for pregnancy success, while depletion of Treg was associated with pregnancy failure^19–22^. Also, in humans, pregnancy complications, like recurrent pregnancy loss and preeclampsia, were found to be associated with lower numbers of Treg^23–26^. Altogether, this suggests that a tightly regulated balance between Th1, Th2, Th17, and Treg cells is required for successful pregnancy.

Although much effort has been put in elucidating how the immune system contributes to pregnancy, particularly in mice, knowledge on human placentation is scarce. Especially little data is available on early implantation and placentation compared to term pregnancy decidual tissue. As local decidual immune regulation is paramount to successful pregnancy, immune phenotypic changes in the uterine immune environment that fit the notion of a well balanced Th1/Th2/Th17/Treg environment might be expected. In the present study, we made a detailed phenotypic and functional analysis of immune cells in both pre-implantation endometrium and in term decidua, with a focus on T cell subsets. We used menstrual blood as a source of endometrial cells because we showed previously, that with respect to cell composition and phenotypic characteristics, menstrual blood is very similar to biopsy-derived material^27^. The results of this study will provide us with a more profound insight into which adaptations of the uterine immune system during pregnancy are important for pregnancy success.

## Results

### The lymphocyte composition of term decidua differs from pre-pregnancy endometrium

**Figure 1:**
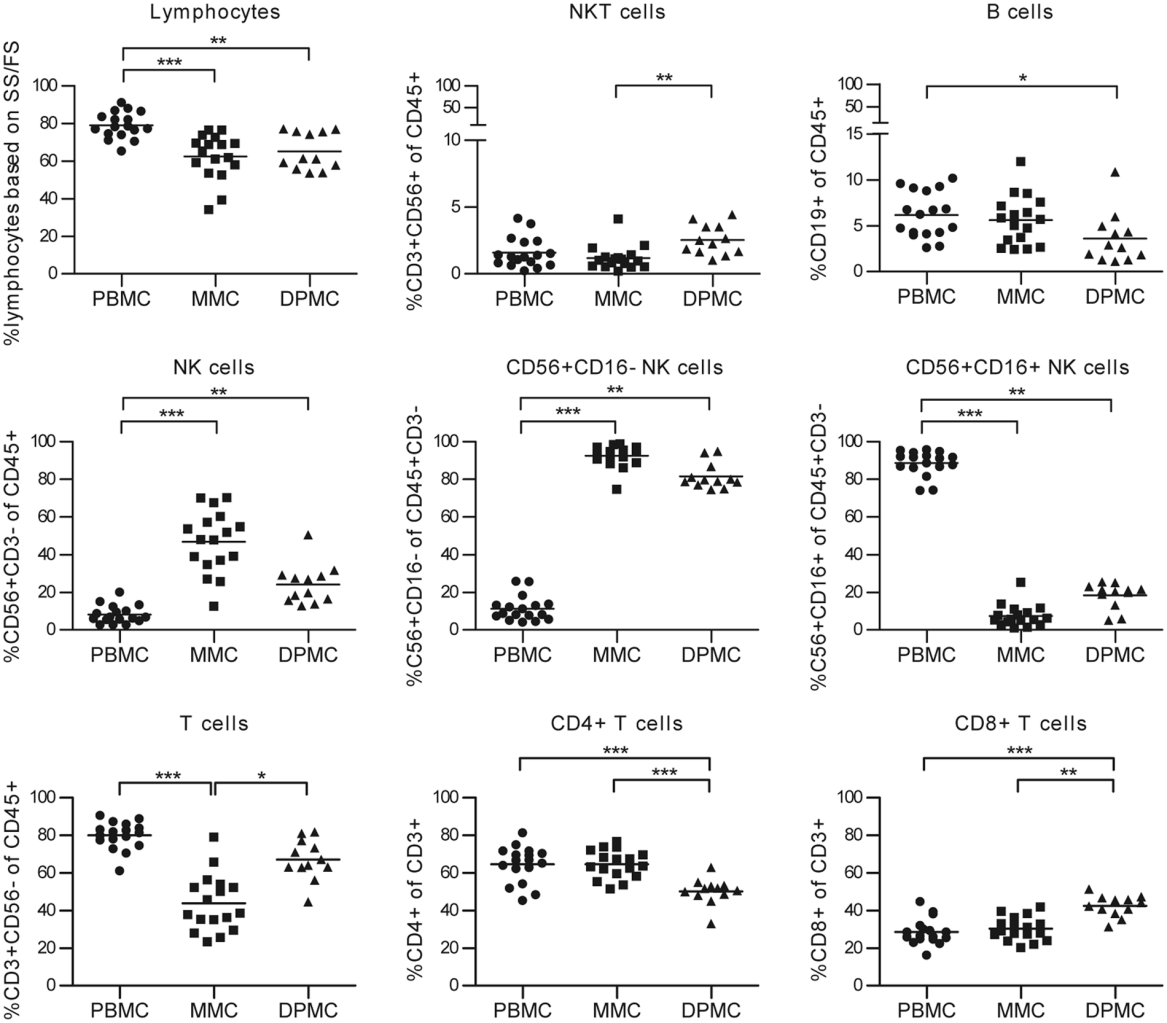
Lymphocyte composition of peripheral blood, menstrual blood, and term decidua. Mononuclear cells were isolated from peripheral blood (PBMC, n = 17), menstrual blood (MMC, n = 17), and decidua parietalis (DPMC, n = 12). Percentages of lymphocytes (CD45^+^) based on side scatter (SS) and forward scatter (FS), T cells (CD56^−^CD3^+^; CD4^+^ and CD8^+^), NKT cells (CD3^+^CD56^+^), NK cells (CD3^−^CD56^+^; CD56^+^CD16^+^ and CD56^+^CD16^−^), and B cells (CD19^+^) were obtained by flow cytometric analysis. *P < 0.05, **P < 0.01, and ***P < 0.001 (lines indicate mean, non-parametric Kruskal-Wallis with Dunns post-hoc test).

Previously, comparisons between endometrium and decidua had to be inferred from separate studies since data on direct comparison of immune cell changes between endometrium and decidua are scarce. The recently designed method, whereby endometrial lymphocytes can be isolated from menstrual blood, allows for easier access to this material and opens up the opportunity to study pre-pregnancy endometrium together with decidual samples in the same set of experiments. Here, we directly compared the immune cell composition of menstrual blood (MMC), term decidua parietalis (DPMC), and peripheral blood (PBMC) mononuclear cells by using flow cytometric analysis (Fig. 1). Since cell yield from decidua basalis was too low for the extensive analysis we did here, maternal lymphocytes in the decidua parietalis are in close contact with chorionic trophoblast cells, and active immune regulation seems to place at the decidua parietalis as well^28, 29^, prompted our decision to opt for isolation of cells from decidua parietalis to study the fetal-maternal interface.

In accordance with previous studies, MMC and DPMC clearly differ from PBMC in percentages of lymphocytes, T cells, NK cells and NKT cells^7–9, 27, 30, 31^, and contained primarily CD56^+^CD16^−^NK cells, while the majority of NK cells in PBMC were CD56^+/−^CD16^+^. In a direct comparison between MMC and DPMC, MMC revealed a higher percentage of NK cells (46.9% ± 16.4% and 24.3% ± 10.6% respectively). MMC (43.9% ± 15.10%) contained a significant lower percentage of T cells, with an increased CD4^+^/CD8^+^ ratio, as compared to DPMC (67.1% ± 10.66%). In addition, MMC contained significantly less NKT cells (1.2% ± 0.9% versus 2.5% ± 1.1%) compared to DPMC. No significant difference in percentages of B cells between MMC and DPMC could be observed. Also, important to note is that MMC and DPMC derived lymphocytes are from mucosal origin since more CD69^+^ and CD103^+^, and less CD62L^+^ T and NK cells can be found compared to PBMC (Supplementary Fig. S2). After showing that MMC and DPMC clearly differ in immune cell composition, we investigated T cells in more depth for differences between pre-pregnancy endometrium and decidua.

### Decidual T cells are more experienced than endometrial T cells

**Figure 2:**
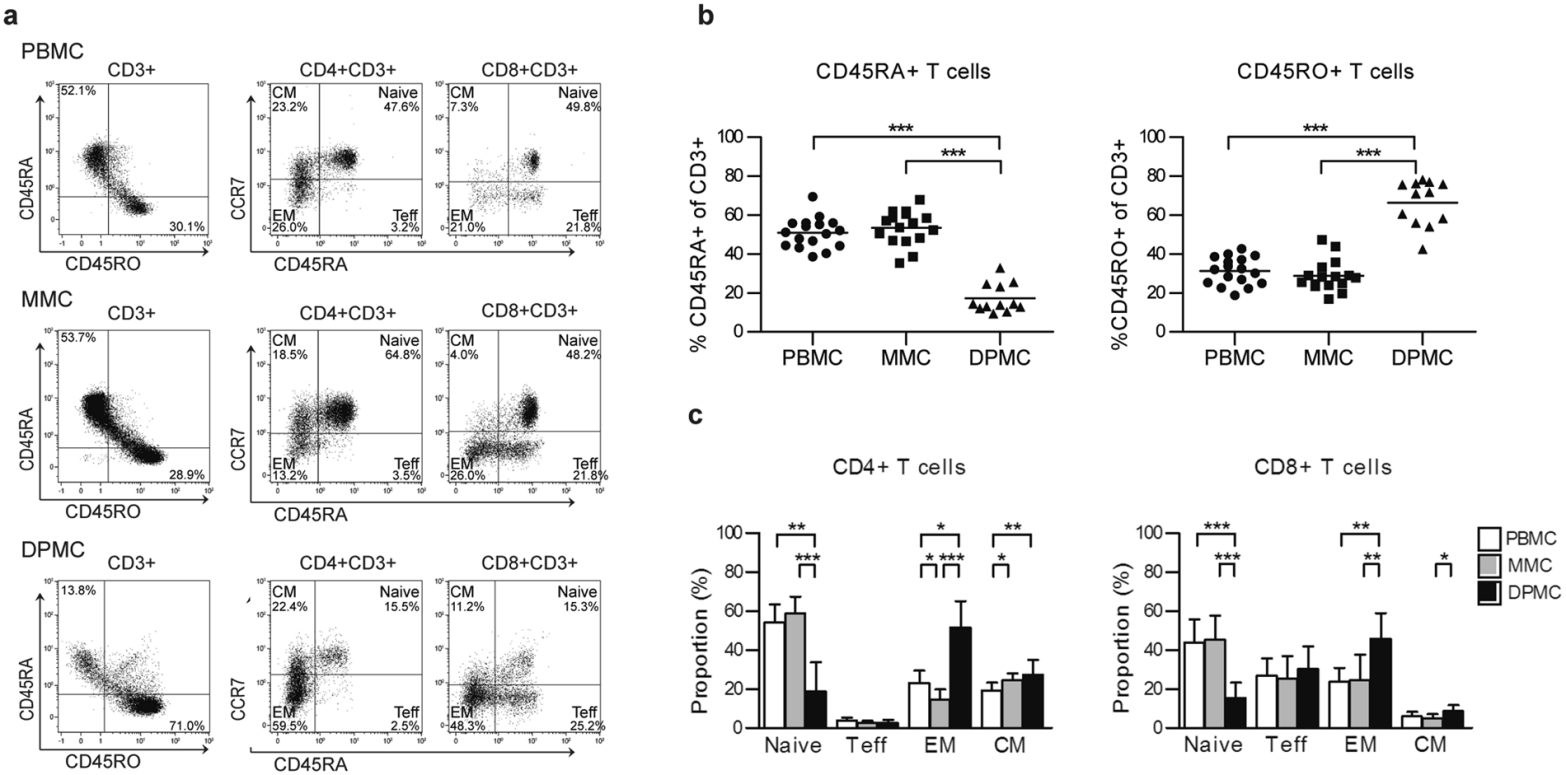
CD4^+^ and CD8^+^ T cells subsets in peripheral blood (PBMC), menstrual blood (MMC) and term decidua parietalis (DPMC). (**a**) Representative staining for CD45RA and CD45RO on CD3^+^ T cells and CD45RA and CCR7 on CD4^+^ and CD8^+^ T cells from PBMC, MMC, and DPMC. (**b**) Expression of CD45RA and CD45RO on CD3^+^ T cells (PBMC n = 17, MMC n = 16, and DPMC n = 12. (**c**) CD4^+^ and CD8^+^ T cells are separated into four subsets based on the expression of CD45RA and CCR7. Naive T cell (CD45RA^+^CCR7^+^); effector T cell, Teff (CD45RA^+^CCR7^−^); effector memory T cell, EM (CD45RA^−^CCR7^−^); central memory T cell, CM (CD45RA^−^CCR7^+^) (PBMC n = 17, MMC n = 11, and DPMC n = 8). Lines indicate mean ± SD. *P < 0.05, **P < 0.01, and ***P < 0.001 (non-parametric Kruskal-Wallis with Dunns post-hoc test).

We next examined endometrial and decidual T cell subsets in more detail to know if the observed phenotype of more differentiated memory T cells in decidua compared to PBMC^32^ would also apply to endometrium. Compared to MMC derived T cells, DPMC derived T cells revealed a significantly lower percentage of CD45RA^+^ (53.4% ± 8.8% and 17.2% ± 7.4%, respectively) and a higher percentage of CD45RO^+^ cells (28.9% ± 8.0% and 66.4% ± 11.7% respectively) (Fig. 2b). In addition, we subdivided CD4^+^ and CD8^+^ T cells into naive T cells (CD45RA^+^CCR7^+^), effector T cells (Teff, CD45RA^+^CCR7^−^), effector memory T cells (EM, CD45RA^−^CCR7^−^), and central memory T cells (CM, CD45RA^−^CCR7^+^). Both CD4^+^ and CD8^+^ T cells present in DPMC were significantly less naive compared to MMC, while a significantly higher percentage of EM and CM T cells was present in DPMC (Fig. 2c). Thus, over the course of pregnancy, decidual T cells appear to acquire an experienced and differentiated phenotype.

To strengthen the claim that endometrial T cells are primed over the course of pregnancy and can differentiate towards a phenotype found in the decidua, we performed *in vitro* experiments whereby endometrial lymphocytes were stimulated with either rhIL-15 or anti-CD3/anti-CD28 mAb microbeads alone, or in combination. The rationale for using IL-15 is because IL-2 is hardly detected in decidua and after implantation, endometrial NK cells start to differentiate as a result of local IL-15 production^9^. After 5 days of culture, the distribution of T cell subsets was measured with flow cytometry in the same way as above. Endometrial T cells stimulated with anti-CD3/anti-CD28 mAb microbeads reveal a similar subset division as found in term decidual T cells, i.e. less naive T cells and more EM and CM T cells compared to control (medium) and day 0 (Supplementary Fig. S3). Whereas stimulation with IL-15 alone was not sufficient to lead to a similar differentiation, IL-15 did appear to have an effect on T cell differentiation when administered together with anti-CD3/anti-CD28 beads and as compared to the bead alone condition, i.e. more EM and less CM T cells. This may suggest that T cells need an additional TCR trigger before they can differentiate towards a more mature phenotype. These *in vitro* data showed that endometrial lymphocytes can be differentiated towards a phenotype reminiscent of term decidual T cells, which lends further support for the notion that over the course of pregnancy decidual T cells appear to acquire an experienced and differentiated phenotype.

### The decidual immune environment is marked by a tolerance signature

**Figure 3:**
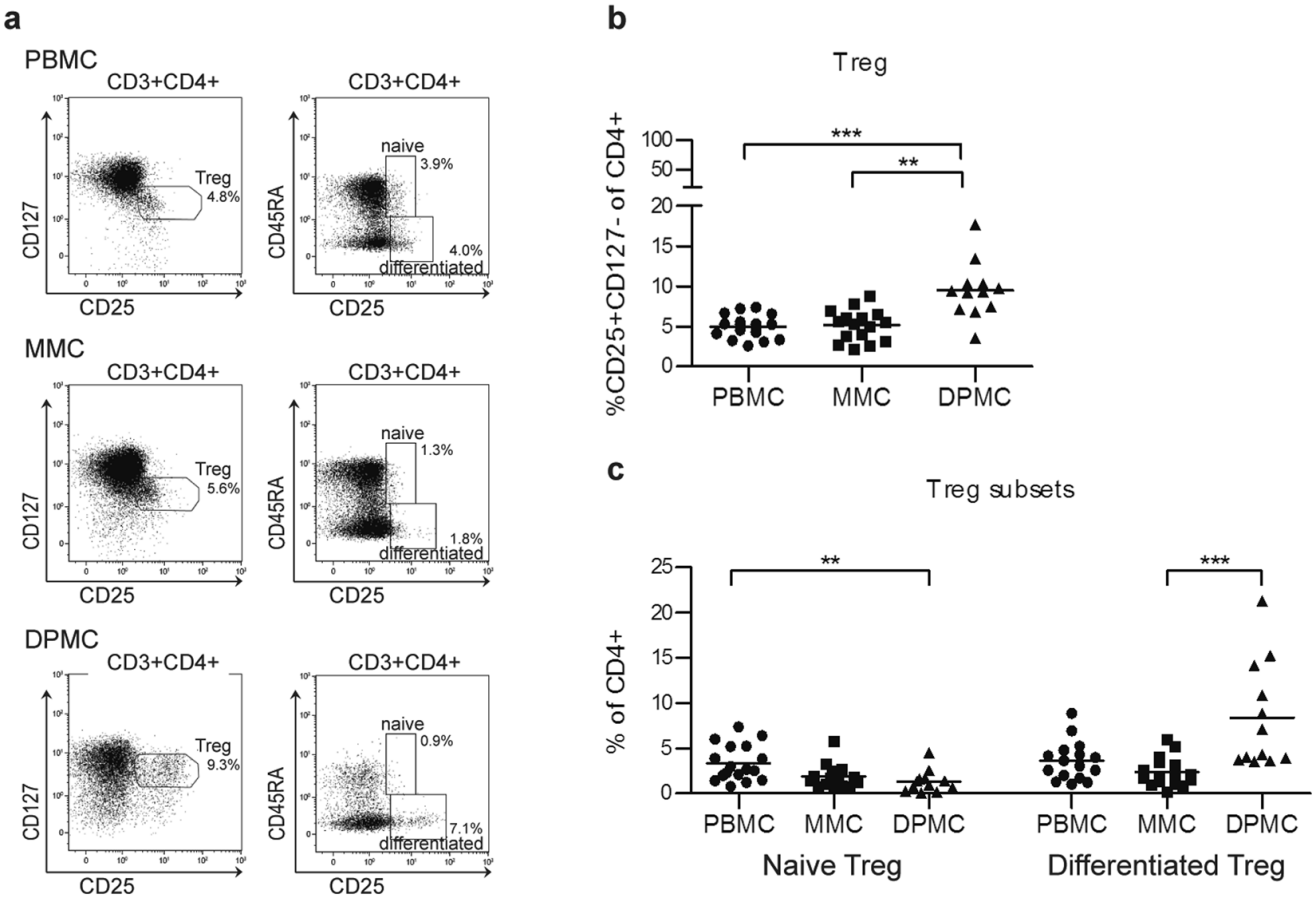
Distribution of regulatory T cells. (**a**) Representative staining of CD25, CD127, and CD45RA on CD4^+^ T cells from peripheral blood (PBMC, n = 17), menstrual blood (MMC, n = 16), and term decidua ﻿parietalis (DPMC, n = 12). (**b**) Percentage of CD25^high^CD127^−^ Treg within CD4^+^ T cells in PBMC, MMC, and DPMC. (**c**) Distribution of naive Treg (CD45RA^+^CD25^+^) and differentiated Treg (CD45RA^−^CD25^++^) in CD4^+^ T cells. **P < 0.01, and ***P < 0.001 (lines indicate mean, non-parametric Kruskal-Wallis with Dunns post-hoc test).

The fetal-maternal interface is the major site where maternal immune cells come into contact with cells of fetal origin. Treg are important for regulation of the decidual immune environment and pregnancy success^19^. DPMC contained significantly more CD4^+^CD25^high^CD127^−^ Treg than MMC (9.5% ± 3.5% versus 5.2% ± 1.9%) (Fig. 3b). We further subdivided Treg based on expression of CD45RA and CD25^33^. DPMC contained significantly more CD4^+^CD25^high^CD45RA^−^ activated and differentiated Treg than MMC (8.4% ± 5.9% versus 2.4% ± 1.6%), while the percentage of CD4^+^CD25^+^CD45RA^+^ naive Treg did not differ significantly (1.3% ± 1.2% versus 1.9% ± 1.3%) (Fig. 3c). Treg percentages in MMC did not differ from PBMC, but DPMC contained significantly less naive Treg compared to PBMC (1.3% ± 1.2% versus 3.4% ± 2.0%). This suggests, that different from the pre-implantation endometrium, the human decidual environment is marked by a immune signature that includes the activation and differentiation of Treg.

### The Th1, Th2, and Th17 cell profile is similar between endometrium and term decidua

**Figure 4:**
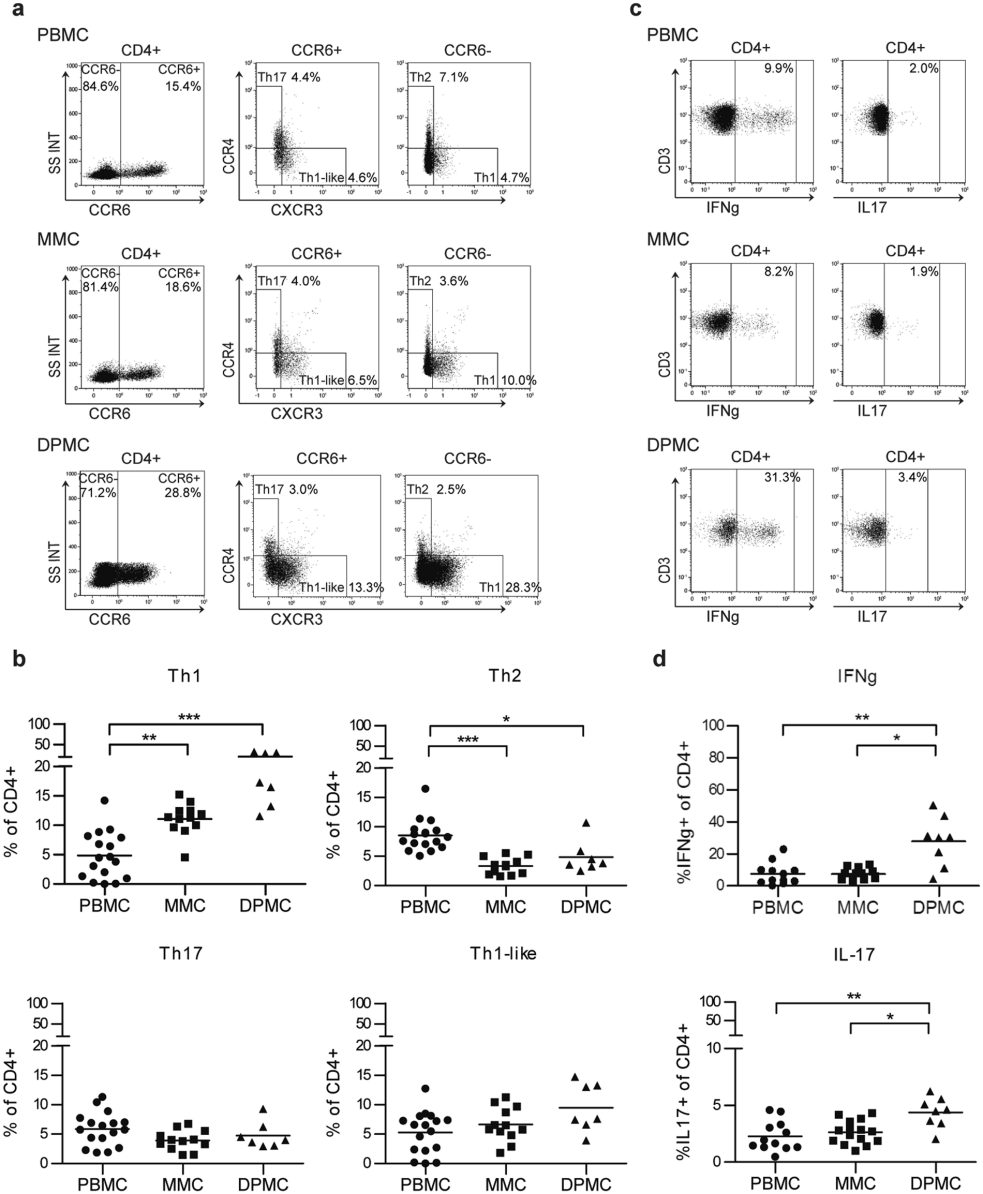
Distribution of CD4^+^ Th cell subsets in peripheral blood (PBMC), menstrual blood (MMC), and term decidua parietalis (DPMC) and production of IFN-γ and IL-17 by CD4^+^ T cells. (**a**,**c**) Representative gating for CCR6, CXCR3, and CCR4 on CD4^+^ T cells and for IFN-γ and IL-17 by CD4^+^ T cells from PBMC, MMC, and DPMC. (**b**) Th1, Th2, Th17, and nonconventional Th1 (Th1-like) CD4^+^ T cells can be classified based on the expression of CCR6, CXCR3, and CCR4^34^. CCR6^−^CXCR3^+^CCR4^−^, Th1; CCR6^+^CXCR3^+^CCR4^−^, Th1-like; CCR6^−^CXCR3^−^CCR4^+^, Th2; CCR6^+^CXCR3^−^CCR4^+^, Th17 (PBMC n = 17, MMC n = 11, DPMC n = 7). (**d**) Production of IFN-γ and IL-17 by CD4^+^ T cells from PBMC (n = 12), MMC (n = 15), and DPMC (n = 7). *P < 0.05, **P < 0.01, and ***P < 0.001 (lines indicate mean, non-parametric Kruskal-Wallis with Dunns post-hoc test).

It is suggested that successful pregnancy requires a delicate Th1/Th2/Th17/Treg balance^14^. To investigate this balance, we classified CD4^+^ cells as Th1, nonconventional Th1 (Th1-like), Th2, or Th17 cells, based on the expression of the chemokine receptors CCR6, CXCR3, and CCR4^34^. Blood Th1-like cells were reported to have a mixed Th1/Th17 phenotype, i.e. production of *RORC* mRNA and IL-17, together with a higher production of *TBX21* mRNA and IFN-γ than conventional Th1 cells, indicating higher Th1 activity^34^. Th cell composition of MMC and DPMC were both different from PBMC, with significantly less Th2, and more Th1 cells in the uterine environment (Fig. 4b). Between MMC and DPMC, percentages of Th1, Th2, Th17, and Th1-like cells did not differ significantly (Fig. 4b). When investigating the intracellular cytokine expression profile after PMA/Ionomycin/Brefeldin stimulation, we observed a higher intrinsic capacity to express IFN-γ and IL-17 by DPMC CD4^+^ T cells (27.8% ± 15.4% and 4.4% ± 1.3% respectively) compared to CD4^+^ T cells from MMC (7.4% ± 3.3% and 2.6% ± 1.0% respectively), and also PBMC (7.5% ± 6.7% and 2.3% ± 1.3% respectively) (Fig. 4d).

In summary, a direct comparison between pre-pregnancy endometrium and term decidua reveals a activated immune signature, with more experienced conventional T cells and Treg. The distribution of Th subsets, defined by chemokine receptor expression patterns, did not differ significantly between endometrium and term decidua. However, the intrinsic capacity to produce IFN-γ and IL-17 was highest in term decidual CD4^+^ T cells, suggesting a possible role for both cytokines in the uterine environment during pregnancy.

## Discussion

Local decidual immune regulation is paramount to successful pregnancy. Previous studies investigating the phenotype of immune cells in endometrium and decidua are fragmented, and limited to comparing immune cells in either endometrium or decidua with their counterparts in peripheral blood^7–9, 27, 30, 31^. Here, we performed a cross-sectional study, directly comparing the immune cell composition and functional capacity of pre-pregnancy endometrium and term decidua using flow cytometric analysis. We show clear differences in immune cell composition, suggestive of immune cell differentiation over the course of pregnancy, i.e. more experienced and less naive T cells and Treg in term decidua compared to pre-pregnancy endometrium. Analysis of the Th1/Th2/Th17/Treg subset composition showed that, while the distribution of Th1, Th2, and Th17 cells did not differ between term decidua and endometrium, there was a preference for Th1 over Th2 cells in the uterine environment compared to peripheral blood.

The role of T cells in pregnancy has been subject to various studies. T cells were shown to be a major cell population in both endometrium and decidua, but their function and antigen-specificity remains poorly defined^13^. We observed mainly effector memory (EM) and central memory (CM) T cells at the end of pregnancy, and they were thus more experienced and differentiated than T cells from pre-pregnancy endometrium. This suggests that over the course of pregnancy, T cells at the fetal-maternal interface may differentiate. After implantation of a blastocyst, endometrium will modify to decidua, and cells of fetal origin will come in to contact with lymphocytes at the maternal-fetal interface. This contact might differentiate endometrial T cells towards a phenotype as seen in decidual tissue, as suggested by our *in vitro* assay. The target specificity of the T cells and exact trigger for this differentiation is unclear, but multiple triggers have been suggested to play a role, including fetal alloantigens like major histocompatibility complex antigens (MHC) (HLA-C in humans)^2, 4, 35^, minor histocompatibility antigens (mHags)^6, 36^, and/or pathogen-derived antigens^37^. In accordance, Tilburgs *et al*. previously reported that in addition to the lower numbers of naive T cells in decidual tissue, mainly EM and few CM CD8^+^ T cells were present in term decidua^32^.

Pregnancy induces local enrichment of Treg at the fetal-maternal interface^38, 39^. These Treg play an important role in tolerance to the semi-allogeneic fetus and pregnancy in mice, since mouse studies showed that depletion of CD25^+^ Treg resulted in gestation failure in allogeneic pregnancies^20–22^. We showed that the percentage of CD4^+^CD25^high^CD127^−^Treg was higher in decidua than in endometrium, suggesting a more tolerogenic environment during pregnancy. As previously reported by our group^27^, no difference in the percentage of Treg in MMC and PBMC samples was observed. The increased percentage of Treg we found in term decidual tissue is comparable to percentages found in other studies^38^. Based on the expression of CD45RA and CD25, human CD4^+^ T cells can be separated into differentiated Treg and naive Treg^33^. We showed that term decidua contained more differentiated Treg than endometrium, while the presence of naive Treg was lower. In peripheral blood, these differentiated Treg were shown to be derived from recently activated naive Treg and were suggested to be the main effectors of suppression^33^. In mice, it was shown that during the course of pregnancy Treg will acquire a protective regulatory memory phenotype to fetal antigen. These memory Treg persist after pregnancy and re-accumulate rapidly in a subsequent pregnancy^40^. A similar phenomenon thus may take place in the uterus during human pregnancy.

Not only Treg, but the overall balance between Th1, Th2, Th17, and Treg cells is suggested to be of relevance for successful pregnancy^14^. Th subsets can be classified by the production of particular cytokines by T cells, or by the expression of different chemokine receptors on T cells^34^. Using a classification based on chemokine receptor expression patterns, we found that both term decidua and pre-pregnancy endometrium held relatively more Th1 cells, and less Th2 cells compared to peripheral blood. Our results thus showed that in the uterine environment, there is a preference of Th1 over Th2 cells. The same trend was also seen in first trimester decidua^41^. This suggests that this preference is already present in pre-pregnancy endometrium and stays over the course of pregnancy until term. Between term decidua and endometrium, the distribution of Th1, Th2, Th17, and Th1-like cells did not significantly differ. The similar Th cell distribution between endometrium and term decidua suggests that the pre-pregnancy endometrium might already be prepared for pregnancy. It can therefore be envisaged that women who experience recurrent miscarriages already have an imbalance in their endometrium affecting a successful pregnancy outcome. For instance, Shimada *et al*.^42^ showed that in the endometrium of women with recurrent miscarriages less CD4^+^IFN-γ^+^ T cells were present. In-depth analysis of the Th1/Th2/Th17/Treg balance in the endometrium of women with fertility issues might give us more insight into the underlying pathogenesis of pregnancy complications and could potentially predict subsequent pregnancy outcome. Interestingly, when looking at the actual intrinsic capacity of CD4^+^ T cells to express IL-17 and IFN-γ intracellular, we found more expression of IFN-γ and IL-17 by decidual T cells compared to peripheral and endometrial CD4^+^ T cells. In first trimester decidua, the percentage of IL-17^+^ T cells was also found to be higher compared to peripheral blood^16, 43^, while the expression of IFN-γ was lower^44^. Although IFN-γ and IL-17 have been related to pregnancy complications, and an excess of inflammation was associated with a negative impact on pregnancy outcome^16, 18, 43, 45, 46^, the presence of IFN-γ^+^ and IL-17^+^ T cells in healthy term decidua suggests that these cytokines may play an important role during pregnancy. IFN-γ for instance, was shown to be essential for implantation, decidual integrity and placental growth in mice^45, 47^. IL-17 plays an important role in host defense against pathogens^15^, but it was also shown that IL-17 increases the production of progesterone by JEG-3 cells and supports the survival, proliferation and invasive capacity of trophoblast cells^48–50^. The presence of normal, balanced levels of IFN-γ and IL-17 could play a role during placentation and/or prevention of intrauterine infection, but this is still far from understood. While we did not show a difference in the capacity of peripheral and endometrial CD4^+^ T cell to express IL-17, Hosseini *et al*.^51^ showed more IL-17^+^CD3^+^ T cells in menstrual blood compared to peripheral blood. This difference can be explained by a different gating approach since we looked at percentage of CD4^+^ T cells, while they looked at CD3^+^ T cells. When we would gate our data in a similar way, we found a similar, although not significant, difference between peripheral and menstrual blood. This can be explained by the higher percentages in our peripheral blood samples (average 4.5%) compared to theirs (average 0.9%). Several reasons could explain this discrepancy: duration of stimulation (4 h vs 6 h) or differences in stimulus and/or concentration used (PMA (12.5 ng/ml)/Ionomycin (500 ng/ml)/Brefeldin A (5 µg/ml) by us versus PMA (25 ng/ml)/Ionomycin (500 ng/ml)/Monensin (1 µM/ml) by them).

A limitation or our study is that we were not able to study decidual tissue at several time-points during pregnancy, to actually show the changes that occur with time. We could only infer this from the data collected from pre-pregnancy endometrium (implantation stage) and term decidua.

In conclusion, we showed that the immune cell composition of pre-pregnancy endometrium differs from term decidua, but with a similar distribution of Th1, Th2, and Th17 cells. At the end of pregnancy, the uterine immune environment appears to be marked by a tolerogenic phenotype with more experienced T cells and Treg with a potential beneficial phenotype. How exactly the phenotype of the uterine immune cells is shaped and how this differentiation is maintained is unclear. Therefore, in follow-up studies, we aim to explore the influence of different immune triggers on immune cells in the uterine environment before and during pregnancy. This may add insight to our understanding of the pathogenesis of pregnancy complications.

## Materials and Methods

### Blood and tissue sampling

Paired peripheral blood and menstrual blood was collected from 17 healthy women with regular menstrual cycles. Hormones can modulate immune cell responses and change the natural menstrual cycle^52, 53^. To avoid any artificial effect on the hormonal balance, none of the menstrual blood donors used any hormonal contraceptives like birth control pill or an intra-uterine device. See Supplementary Table S1 for donor characteristics. 10 ml of peripheral blood was collected in ACD-A tubes. Menstrual blood was collected during the first 36 hours of menstruation using a menstrual cup (Femmecup Ltd, London, UK). Every 12 hours, the sample was decanted from the cup in a 30 ml tube containing 8 ml 10% human pooled serum (HPS) medium [RPMI 1640 medium supplemented with pyruvate (1 mM), glutamax (2 mM), penicillin (100 U/ml), streptomycin (100 mg/ml) (Thermo Fisher Scientific, Waltham, USA), 10% HPS (manufactured in-house), and 0.3% sodium citrate (Merck, Darmstadt, Germany)] and stored at room temperature. After 1.5 days, three tubes containing different fractions of menstrual blood were processed immediately to assess lymphocyte composition. Decidua parietalis, the maternal side of the fetal-maternal interface, was obtained from 19 healthy women after uncomplicated term pregnancy. Decidual samples were obtained after delivery by planned elective cesarean section and processed immediately. The study was approved by the institutional review board (Commissie Mensgebonden Onderzoek region Arnhem-Nijmegen, CMO nr. 2009/004) and was performed in accordance with the relevant guidelines and regulations. Samples were obtained from each participant upon written informed consent.

### Isolation of lymphocytes

One ml of peripheral blood was lysed with 25 ml lysis buffer [NH_4_CL + KHCO_3_/Na_4_EDTA (Merck, Darmstadt, Germany) diluted in H_2_O (Versol, Lyon, France)] for 10 min and washed 3x times with PBS (Braun, Melsungen, Germany). These cells were used for surface staining. For intracellular staining, peripheral blood mononuclear cells (PBMC) were isolated by means of density gradient centrifugation (Lymphoprep; Axis-Shield PoC AS, Oslo, Norway). After isolation, cells were washed twice with PBS. Menstrual blood was washed with PBS and passed through a 70 µm cell strainer (Falcon, Durham, USA) to remove clots and mucus. Granulocytes were depleted by use of a granulocyte depletion kit according to the manufacturer’s instructions (STEMCELL Technologies, Vancouver, Canada). After isolation, cells were washed twice with PBS containing 2% HPS. Decidua parietalis was collected as described previously^38^. Briefly, after removing the amnion, the decidua parietalis was carefully scraped from the chorion. The obtained tissue was washed thoroughly in PBS before mincing with scissors. The resulting pulp was washed again until the supernatant became transparent. The tissue was enzymatically incubated with 1% collagenase I (Gibco Life Technologies, Waltham, USA) and 1% DNAse (Roche Diagnostics, Risch-Rotkreuz, Switzerland) in a water bath at 37 °C while shaking for 60 minutes. After washing with RPMI medium, the suspension was passed through a 70 μm cell strainer (Greiner, Frickenhausen, Germany) and washed again with RPMI. Lymphocytes were obtained after density gradient centrifugation (Lymphoprep). Analysis was done immediately on fresh material to exclude the influence of cryopreservation on the expression of certain markers. For optimal analysis of the chemokine receptors CD183 (CXCR3), CD194 (CCR4), and CD196 (CCR6) on decidual cells, cells were put to rest at 37 °C in a humidified 5% CO_2_ incubator for 16 hours before staining for flow cytometry. Typically 93%, 95%, and 81% of respectively peripheral, menstrual, and decidual lymphocytes were viable cells (Supplementary Fig. S1).

### Flow cytometry

Samples were phenotypically analyzed using the 10-color NaviosTM flow cytometer (Beckman Coulter, Fullerton, CA, USA). Briefly, cells were washed twice with PBS + 0.2% bovine serum albumin (BSA; Sigma-Aldrich, St. Louis, USA) and labeled for 20 min at RT in the dark with the fluorochrome-conjugated mAbs of interest. Samples were washed twice with PBS + 0.2% BSA. For cell surface staining of B cells, monocytes/macrophages, T cells, Treg, NKT cells and NK cells, the following conjugated mAbs were used: CD3-PE/ECD/PB (Beckman Coulter; UCHT1), CD4-PC5.5/PB (Beckman Coulter; 13B8.2), CD4-AF700 (eBioscience, San Diego, USA; RPA-T4), CD8-APC-AF700/APC-AF750 (Beckman Coulter; B9.11), CD14-ECD (Beckman Coulter; RMO52), CD16^−^FITC (Beckman Coulter; 3G8), CD19-APC-AF750 (Beckman Coulter; J3-119), CD25-PC7/APC (BD Biosciences, New Jersey, USA; M-A251 and 2A3), CD45-KO (Beckman Coulter; J33), CD45RA-FITC/ECD (Beckman Coulter; ALB11 and 2H4LDH11LDB9), CD45RO-ECD (Beckman Coulter; UCHL1), CD56-APC (Beckman Coulter; N901), CD62L-FITC/ECD (eBiosience and Beckman Coulter; DREG-56), CD69-PE (Beckman Coulter; TP1.55.3), CD103-FITC (eBioscience; B-Ly7), CD127-APC-AF700 (Beckman Coulter; R34.34), CD183-PC5.5 (CXCR3; Biolegend, San Diego, USA; G025H7), CD194-PC7 (CXCR4; BD Biosciences; 1G1), CD196-PE (CCR6; BD Biosciences; 11A9), CD197-BV421 (CCR7; BioLegend; G043H7), and Fixable Viability Dye-eFluor780 (eBioscience). For intracellular staining, samples were permeabilized and fixed according to manufacturer’s instructions (eBioscience). Cells were incubated with the conjugated mAbs of interest for 30 min at 4 °C in the dark. The following conjugated mAbs for intracellular staining were used: IFN-γ-PC7 (4 S.B3) and IL-17-APC-AF780 (eBioscience; eBio64DEC17). A minimum of 200.000 cells per staining was applied. Fluorescence minus one (FMO) and isotype controls were used for gate settings. The data were analyzed using Kaluza V1.1 software (Beckman Coulter). A typical gating strategy used for analysis of T cells and NK cells is depicted in Supplementary Fig. S1.

### Functional analysis

Peripheral blood (PBMC), menstrual blood (MMC), and decidua parietalis (DPMC) mononuclear cells were stimulated with phorbol-12-myristate-13-acetate (PMA), ionomycin and brefeldin A (respectively, 12.5 ng/ml, 500 ng/ml and 5 µg/ml; Sigma-Aldrich, St. Louis, USA) for 4 hours at 37 °C in a humidified 5% CO_2_ incubator. Functionality was determined by measuring the intracellular production of IFN-γ and IL-17 by flow cytometry as described above.

### Differentiation assay

MMC were stimulated *in vitro* with medium as a control, with anti-CD3/anti-CD28 mAb-coated microbeads in a 1:10 bead-to-cell ratio (Invitrogen, Bleiswijk, The Netherlands) alone, rhIL-15 (100 ng/ml; Gibco Life Technologies) alone, and beads together with rhIL-15 in 96-well U-bottom plates. The rationale for using IL-15 is because IL-2 is hardly detected in decidua and after implantation, endometrial NK cells start to differentiate as a result of local IL-15 production^9^. After 5 days of culture at 37 °C in a humidified 5% CO_2_ incubator, cells were harvested and the presence of T cell subsets and differentiation was measured with flow cytometry.

### Statistical analysis

Statistical analyses were performed using GraphPad Prism 5 (Graphpad software Inc., La Jolla, CA, USA). Non-parametric Kruskal-Wallis with Dunns post-hoc test was performed to compare PBMC, MMC, and DPMC samples. Statistical significance was denoted as values of *P* < 0.05. All indicated values are mean percentages ± SD.

## Electronic supplementary material

Supplementary information

## References

[1] Moffett A, Loke C (2006). Immunology of placentation in eutherian mammals. Nature reviews. Immunology.

[2] Tilburgs T (2009). Fetal-maternal HLA-C mismatch is associated with decidual T cell activation and induction of functional T regulatory cells. Journal of reproductive immunology.

[3] Tilburgs T (2015). Human HLA-G+ extravillous trophoblasts: Immune-activating cells that interact with decidual leukocytes. Proceedings of the National Academy of Sciences of the United States of America.

[4] Lissauer D, Piper K, Goodyear O, Kilby MD, Moss PA (2012). Fetal-specific CD8+ cytotoxic T cell responses develop during normal human pregnancy and exhibit broad functional capacity. Journal of immunology.

[5] van Halteren AG (2009). Naturally acquired tolerance and sensitization to minor histocompatibility antigens in healthy family members. Blood.

[6] Holland OJ (2012). Minor histocompatibility antigens are expressed in syncytiotrophoblast and trophoblast debris: implications for maternal alloreactivity to the fetus. The American journal of pathology.

[7] Trundley A, Moffett A (2004). Human uterine leukocytes and pregnancy. Tissue Antigens.

[8] Lukassen HG (2004). Hormonal stimulation for IVF treatment positively affects the CD56bright/CD56dim NK cell ratio of the endometrium during the window of implantation. Molecular human reproduction.

[9] Manaster I (2008). Endometrial NK cells are special immature cells that await pregnancy. Journal of immunology.

[10] Hanna J (2006). Decidual NK cells regulate key developmental processes at the human fetal-maternal interface. Nature medicine.

[11] Tilburgs T (2009). Expression of NK cell receptors on decidual T cells in human pregnancy. Journal of reproductive immunology.

[12] Saito S (1992). Expression of activation antigens CD69, HLA-DR, interleukin-2 receptor-alpha (IL-2R alpha) and IL-2R beta on T cells of human decidua at an early stage of pregnancy. Immunology.

[13] Nancy P, Erlebacher A (2014). T cell behavior at the maternal-fetal interface. The International journal of developmental biology.

[14] Saito S, Nakashima A, Shima T, Ito M (2010). Th1/Th2/Th17 and regulatory T-cell paradigm in pregnancy. American journal of reproductive immunology.

[15] Crome SQ, Wang AY, Levings MK (2010). Translational mini-review series on Th17 cells: function and regulation of human T helper 17 cells in health and disease. Clin Exp Immunol.

[16] Wang WJ (2010). Increased prevalence of T helper 17 (Th17) cells in peripheral blood and decidua in unexplained recurrent spontaneous abortion patients. Journal of reproductive immunology.

[17] Nakashima A (2010). Accumulation of IL-17-positive cells in decidua of inevitable abortion cases. American journal of reproductive immunology.

[18] Ito M (2010). A role for IL-17 in induction of an inflammation at the fetomaternal interface in preterm labour. Journal of reproductive immunology.

[19] Ruocco MG, Chaouat G, Florez L, Bensussan A, Klatzmann D (2014). Regulatory T-cells in pregnancy: historical perspective, state of the art, and burning questions. Frontiers in immunology.

[20] Aluvihare VR, Kallikourdis M, Betz AG (2004). Regulatory T cells mediate maternal tolerance to the fetus. Nature immunology.

[21] Chen T (2013). Self-specific memory regulatory T cells protect embryos at implantation in mice. Journal of immunology.

[22] Shima T (2010). Regulatory T cells are necessary for implantation and maintenance of early pregnancy but not late pregnancy in allogeneic mice. Journal of reproductive immunology.

[23] Sasaki Y (2004). Decidual and peripheral blood CD4+CD25+ regulatory T cells in early pregnancy subjects and spontaneous abortion cases. Molecular human reproduction.

[24] Yang H (2008). Proportional change of CD4+CD25+ regulatory T cells in decidua and peripheral blood in unexplained recurrent spontaneous abortion patients. Fertility and sterility.

[25] Santner-Nanan B (2009). Systemic increase in the ratio between Foxp3+ and IL-17-producing CD4+ T cells in healthy pregnancy but not in preeclampsia. Journal of immunology.

[26] Inada K (2013). Characterization of regulatory T cells in decidua of miscarriage cases with abnormal or normal fetal chromosomal content. Journal of reproductive immunology.

[27] van der Molen RG (2014). Menstrual blood closely resembles the uterine immune micro-environment and is clearly distinct from peripheral blood. Human reproduction.

[28] Sindram-Trujillo A, Scherjon S, Kanhai H, Roelen D, Claas F (2003). Increased T-cell activation in decidua parietalis compared to decidua basalis in uncomplicated human term pregnancy. American journal of reproductive immunology.

[29] Sindram-Trujillo AP (2003). Differential distribution of NK cells in decidua basalis compared with decidua parietalis after uncomplicated human term pregnancy. Human immunology.

[30] Tsuda H (2001). Characterization of NKT cells in human peripheral blood and decidual lymphocytes. American journal of reproductive immunology.

[31] Sabbaj S, Hel Z, Richter HE, Mestecky J, Goepfert PA (2011). Menstrual blood as a potential source of endometrial derived CD3+ T cells. PloS one.

[32] Tilburgs T (2010). Human decidual tissue contains differentiated CD8+ effector-memory T cells with unique properties. Journal of immunology.

[33] Miyara M (2009). Functional delineation and differentiation dynamics of human CD4+ T cells expressing the FoxP3 transcription factor. Immunity.

[34] Acosta-Rodriguez EV (2007). Surface phenotype and antigenic specificity of human interleukin 17-producing T helper memory cells. Nature immunology.

[35] Petersdorf EW (1997). Association of HLA-C disparity with graft failure after marrow transplantation from unrelated donors. Blood.

[36] Goulmy E (1996). Human minor histocompatibility antigens. Current opinion in immunology.

[37] Tilburgs T, Strominger JL (2013). CD8+ effector T cells at the fetal-maternal interface, balancing fetal tolerance and antiviral immunity. American journal of reproductive immunology.

[38] Tilburgs T (2006). Differential distribution of CD4(+)CD25(bright) and CD8(+)CD28(−) T-cells in decidua and maternal blood during human pregnancy. Placenta.

[39] Tilburgs T (2008). Evidence for a selective migration of fetus-specific CD4+CD25bright regulatory T cells from the peripheral blood to the decidua in human pregnancy. Journal of immunology.

[40] Rowe JH, Ertelt JM, Xin L, Way SS (2012). Pregnancy imprints regulatory memory that sustains anergy to fetal antigen. Nature.

[41] Mjosberg J, Berg G, Jenmalm MC, Ernerudh J (2010). FOXP3+ regulatory T cells and T helper 1, T helper 2, and T helper 17 cells in human early pregnancy decidua. Biol Reprod.

[42] Shimada S (2004). No difference in natural killer or natural killer T-cell population, but aberrant T-helper cell population in the endometrium of women with repeated miscarriage. Human reproduction.

[43] Nakashima A (2010). Circulating and decidual Th17 cell levels in healthy pregnancy. American journal of reproductive immunology.

[44] Saito S (1999). Distribution of Th1, Th2, and Th0 and the Th1/Th2 cell ratios in human peripheral and endometrial T cells. American journal of reproductive immunology.

[45] Murphy SP (2009). Interferon gamma in successful pregnancies. Biol Reprod.

[46] Makhseed M (2003). Pro-inflammatory maternal cytokine profile in preterm delivery. American journal of reproductive immunology.

[47] Ashkar AA, Di Santo JP, Croy BA (2000). Interferon gamma contributes to initiation of uterine vascular modification, decidual integrity, and uterine natural killer cell maturation during normal murine pregnancy. The Journal of experimental medicine.

[48] Pongcharoen S (2007). Interleukin-17 expression in the human placenta. Placenta.

[49] Pongcharoen S, Supalap K (2009). Interleukin-17 increased progesterone secretion by JEG-3 human choriocarcinoma cells. American journal of reproductive immunology.

[50] Wu HX, Jin LP, Xu B, Liang SS, Li DJ (2014). Decidual stromal cells recruit Th17 cells into decidua to promote proliferation and invasion of human trophoblast cells by secreting IL-17. Cellular & molecular immunology.

[51] Hosseini S (2016). A shift in the balance of T17 and Treg cells in menstrual blood of women with unexplained recurrent spontaneous abortion. Journal of reproductive immunology.

[52] Huijbregts RP (2013). Hormonal contraception and HIV-1 infection: medroxyprogesterone acetate suppresses innate and adaptive immune mechanisms. Endocrinology.

[53] Auerbach L, Hafner T, Huber JC, Panzer S (2002). Influence of low-dose oral contraception on peripheral blood lymphocyte subsets at particular phases of the hormonal cycle. Fertility and sterility.

