# Supplementary material for: Human uterine lymphocytes acquire a more experienced and tolerogenic phenotype during pregnancy
Source: Sci Rep. 2017 Jun 6;7:2884. doi: 10.1038/s41598-017-03191-0 (PMC5460245; doi:10.1038/s41598-017-03191-0)
Supplement: Supplementary file 1 — Supplementary information [file 41598_2017_3191_MOESM1_ESM.pdf]

Human uterine lymphocytes acquire a more experienced and tolerogenic phenotype during pregnancy

Dorien Feyaerts[1], Marilen Benner[1], Bram van Cranenbroek[1], Olivier W.H. van der Heijden[2], Irma Joosten[1], Renate G. van der Molen[1]*

[1]Department of Laboratory Medicine, Laboratory of Medical Immunology, Radboud University Medical Center, Nijmegen, 6500 HB, The Netherlands

[2]Department of Obstetrics and Gynaecology, Radboud University Medical Center, Nijmegen, 6500 HB, The Netherlands

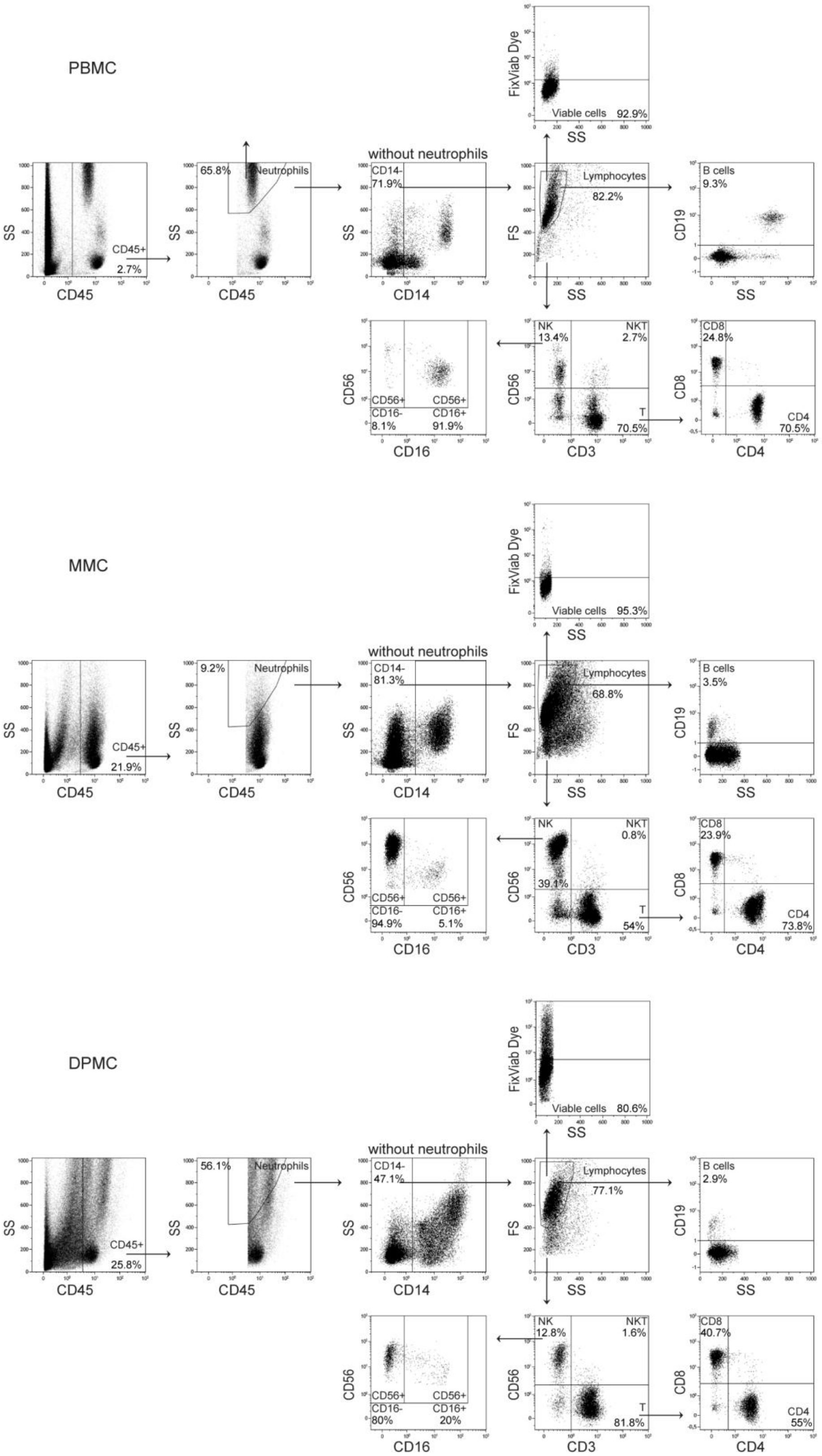

Supplementary Figure S1

Representative gating strategy for immunophenotyping of peripheral blood (PBMC), menstrual blood (MMC), and term decidua (DPMC). Gate settings for PBMC were copied to menstrual and decidual samples. Leukocytes were first gated on CD45 positivity. Neutrophils and CD14$^+$ cells were then

excluded. The remaining dot plots represent the gating strategy for lymphocytes (FS-SS scatter), B cells ($CD19^{+}$), NK cells ($CD3^{-}CD56^{+}$), NKT cells ($CD3^{+}CD56^{+}$), T cells ($CD3^{+}CD56^{-}$), $CD4^{+}$ T cells ($CD3^{+}CD56^{-}CD4^{+}$), $CD8^{+}$ T cells ($CD3^{+}CD56^{-}CD8^{+}$), and viable lymphocytes ($FixViabDye^{-}$).

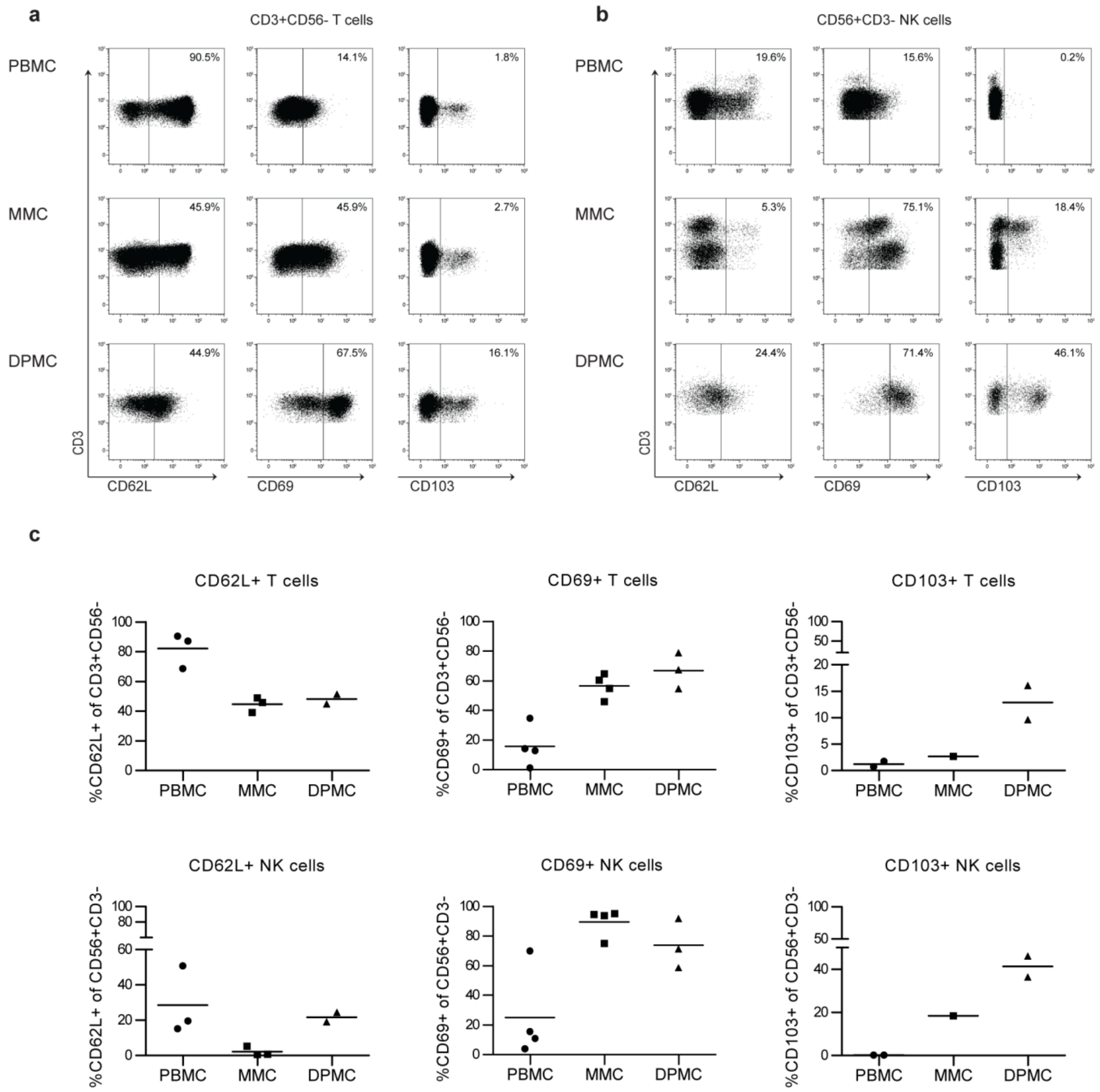

Supplementary Figure S2

Uterine lymphocytes are from mucosal origin. (**a**) Representative FACS plots for the expression of CD62L, CD69, and CD103 on T cells (CD3+CD56-) from peripheral blood (PBMC), menstrual blood (MMC), and decidua (DPMC). (**b**) Representative FACS plots for the expression of CD62L, CD69, and CD103 on NK cells (CD56+CD3-) from PBMC, MMC, and DPMC. (c) Percentage CD62L$^+$, CD69$^+$, and CD103$^+$ T cells and NK cells in PBMC (n=2-4), MMC (n=1-4), and DPMC (n=2-3).

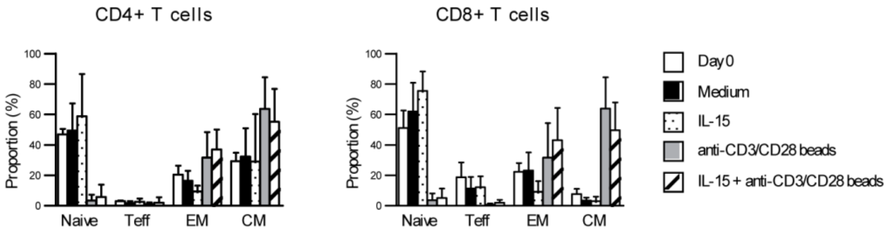

Supplementary Figure S3

CD4^{+} and CD8^{+} T cell subsets after in vitro differentiation of endometrial lymphocytes towards a decidual-like phenotype (n=4). Naive T cell (CD45RA^{+}CCR7^{+}); effector T cell, Teff (CD45RA^{+}CCR7^{-}); effector memory T cell, EM (CD45RA^{-}CCR7^{-}); central memory T cell, CM (CD45RA^{-}CCR7^{+}). Lines indicate mean ± SD.

Supplementary Table S1 Donor characteristics

Variable	Menstrual and peripheral blood donors (n=17)	Placenta donors (n=19) (information not available for 3 donors)
Age, median (range)	30 (19-46)	31 (23-37)
Contraceptives	0/17 (100%)	NA
Previous pregnancy	9/17 (53%)	15/16 (94%)
Previous miscarriage	6/17 (35%)	4/16 (25%)
Length of menstrual cycle, median (range)	28 (22-35)	NA
C-section	NA	19/19 (100%)
Duration of pregnancy, median (range)	NA	38 weeks (38-40 weeks)
Natural conception	NA	15/16 (94%; 1 intrauterine insemination)
Birth weight, median (range)	NA	3340 (2948-4325)

NA, not applicable

Supplementary Table S2 Mean% ± SD is provided for lymphocyte populations in the three study groups as used in the figures.

	Peripheral blood	Menstrual blood	Decidua
Lymphocytes	79.1±6.9	62.5±12.1	65.2±9.8
T cells ($CD3^+CD56^-$)	80.0±7.3	43.9±15.1	67.1±10.6
$CD45RA^+$	50.9±7.7	53.4±8.8	17.2±7.4
$CD45RO^+$	31.2±7.1	28.9±8.0	66.4±11.7
$CD4^+$ T cells	64.6±9.7	64.7±7.4	50.3±7.0
Naive ($CD45RA^+CCR7^+$)	54.1±9.4	58.7±8.6	18.6±15.0
Teff ($CD45RA^+CCR7^-$)	3.8±1.5	2.6±0.9	2.5±1.6
EM ($CD45RA^-CCR7^-$)	23.0±6.5	14.4±5.3	51.6±13.4
CM ($CD45RA^-CCR7^+$)	19.1±4.1	24.3±3.8	27.4±7.6
Treg ($CD25^{high}CD127^-$)	5.0±1.4	5.1±1.9	9.5±3.5
Naïve Treg ($CD45RA^+CD25^+$)	3.4±2.0	1.9±1.3	1.3±1.2
Differentiated Treg ($CD45RA^-CD25^{++}$)	3.7±2.1	2.4±1.6	8.4±5.9
Th1 ($CCR6^-CXCR3^+CCR4^-$)	4.8±4.0	11.1±2.7	21.3±8.7
Th2 ($CCR6^-CXCR3^-CCR4^+$)	8.6±2.7	3.3±1.4	4.9±2.8
Th17 ($CCR6^+CXCR3^-CCR4^+$)	5.9±2.8	3.9±1.7	4.8±2.3
Th1-like ($CCR6^-CXCR3^+CCR4^-$)	5.3±3.6	6.6±2.9	9.5±4.1
$IL\text{-}17^+$ T cells	2.3±1.3	2.6±1.0	4.4±1.3
$IFN\text{-}\gamma^+$ T cells	7.5±6.7	7.4±3.3	27.8±15.4
$CD8^+$ T cells	28.6±6.9	30.3±6.3	42.5±5.6
Naive ($CD45RA^+CCR7^+$)	43.6±11.9	45.3±12.6	15.2±8.0
Teff ($CD45RA^+CCR7^-$)	26.6±9.1	25.3±11.5	30.4±11.5
EM ($CD45RA^-CCR7^-$)	23.7±7.0	24.5±13.1	45.8±13.2
CM ($CD45RA^-CCR7^+$)	6.0±2.2	5.0±1.9	8.7±3.0
NK cells ($CD56^+CD3^-$)	8.2±4.8	46.9±16.4	24.3±10.6
$CD56^+CD16^-$ NK cells	11.4±6.7	92.5±5.8	81.6±6.8
$CD56^+CD16^+$ NK cells	88.7±6.7	7.5±5.8	18.5±6.8
MFI CD56 of $CD56^+CD16^-$ NK cells	52.6±15.5	81.2±19.0	18.1±10.8
NKT cells ($CD3^+CD56^+$)	1.6±1.1	1.2±0.9	2.5±1.1
B cells ($CD19^+$)	6.2±2.5	5.6±2.7	3.6±2.8
